# *p*-Cymene Promotes Its Catabolism through the *p*-Cymene and the *p*-Cumate Pathways, Activates a Stress Response and Reduces the Biofilm Formation in *Burkholderia xenovorans* LB400

**DOI:** 10.1371/journal.pone.0169544

**Published:** 2017-01-10

**Authors:** Loreine Agulló, María José Romero-Silva, Mirian Domenech, Michael Seeger

**Affiliations:** 1 Molecular Microbiology and Environmental Biotechnology Laboratory, Department of Chemistry & Center for Nanotechnology and Systems Biology, Centro de Biotecnología, Universidad Técnica Federico Santa María, Valparaíso, Chile; 2 Departamento de Microbiología Molecular y Biología de las Infecciones, Centro de Investigaciones Biológicas (CSIC), Madrid, Spain; MJP Rohilkhand University, INDIA

## Abstract

*p*-Cymene is an aromatic terpene that is present in diverse plant species. The aims of this study were to study the *p*-cymene metabolism in the model aromatic-degrading bacterium *Burkholderia xenovorans* LB400, and its response to *p*-cymene. The catabolic *p*-cymene (*cym*) and *p*-cumate *(cmt)* genes are clustered on the LB400 major chromosome. *B*. *xenovorans* LB400 was able to grow on *p*-cymene as well as on *p*-cumate as a sole carbon and energy sources. LB400 growth attained higher cell concentration at stationary phase on *p*-cumate than on *p*-cymene. The transcription of the key *cymAb* and *cmtAb* genes, and *p*-cumate dioxygenase activity were observed in LB400 cells grown on *p*-cymene and on *p*-cumate, but not in glucose-grown cells. Diverse changes on LB400 proteome were observed in *p*-cymene-grown cells compared to glucose-grown cells. An increase of the molecular chaperones DnaK, GroEL and ClpB, the organic hydroperoxide resistance protein Ohr, the alkyl hydroperoxide reductase AhpC and the copper oxidase CopA during growth on *p*-cymene strongly suggests that the exposure to *p*-cymene constitutes a stress condition for strain LB400. Diverse proteins of the energy metabolism such as enolase, pyruvate kinase, aconitase AcnA, succinyl-CoA synthetase beta subunit and ATP synthase beta subunit were induced by *p*-cymene. Electron microscopy showed that *p*-cymene-grown cells exhibited fuzzy outer and inner membranes and an increased periplasm. *p*-Cymene induced diverse membrane and transport proteins including the *p*-cymene transporter CymD. Biofilm formation was reduced during growth in *p*-cymene in strain LB400 compared to glucose-grown cells that may be associated with a decrease of diguanylate cyclase protein levels. Overall, these results indicate active *p*-cymene and *p*-cumate catabolic pathways in *B*. *xenovorans* LB400. In addition, this study showed that *p*-cymene activated a stress response in strain LB400 and reduced its biofilm formation.

## Introduction

A large number of aromatic compounds derived from plants and microorganisms are present in surface soil and the rhizosphere [1]. Alkyl-substituted hydrocarbons are components of fossil fuels such as petroleum and coal, and as natural products [2,3]. *p*-Cymene is an aromatic terpene that has been identified in volatile oils from more than 100 plant species, including eucalyptus, cumin, cypress, star anise and cinnamon [4]. *p*-Cymene is degraded via 2,3-dihydroxy-*p*-cumate into tricarboxylic acid (TCA) cycle precursors by *Pseudomonas putida* strains F1 and KL47 [5,6,7]. *Rhodococcus* sp. T104 and *Rhodopseudomonas palustris* are able to degrade *p*-cumate but not *p*-cymene [8,9]. The catabolism of *p*-cymene has been characterized extensively in *P*. *putida* strains F1 and KL47 [5,6,7]. *p*-Cymene is transformed into *p*-cumate by the *p*-cymene peripheral pathway that involves three metabolic steps catalyzed by a two-component monooxygenase, an alcohol dehydrogenase, and an aldehyde dehydrogenase [6,10]. The *p*-cymene peripheral pathway is similar to toluene/xylene and methylbenzoate catabolic pathways from *Pseudomonas* [6]. The degradation is initiated by *p*-cymene monooxygenase CymA that oxygenates the *p*-cymene methyl group in the presence of reduced nicotinamide adenine dinucleotide (NADH) producing *p*-cumic alcohol. *p*-Cumic alcohol is oxidized through the alcohol dehydrogenase CymB and the aldehyde dehydrogenase CymC into *p*-cumate. The degradation of *p*-cumate has been reported in *P*. *putida* strains F1 and KL47 and *R*. sp. strain T104 [5,7,9]. *p*-Cumate is converted via 2,3-dihydroxy-*p*-cumate through eight steps into isobutyrate, pyruvate and acetyl-CoA [5,9,11]. The *cmt* genes from *P*. *putida* F1 were cloned in *P*. *aeruginosa* PAO1 and *Escherichia coli* JM109 and the enzymatic activities of the gene products were characterized [5]. The genes *cmtAaAbAc* and *cmtAd* encode the four *p*-cumate-2,3-dioxygenase subunits: large and small subunits of the terminal dioxygenase, ferredoxin reductase and ferredoxin. *p*-Cumate-2,3-dioxygenase hydroxylates *p*-cumate into 2,3-dihydroxy-2,3-dihydro-*p*-cumate. The *cmtB* gene encodes the 2,3-dihydroxy-2,3-dihydro-*p*-cumate dehydrogenase that converts 2,3-dihydroxy-2,3-dihydro-*p*-cumate into 2,3-dihydroxy-*p*-cumate. The 2,3-dihydroxy-*p*-cumate central catabolic pathway starts with an extradiol cleavage by 2,3-dihydroxy-*p*-cumate 3,4-dioxygenase CmtC. CmtC opens the aromatic ring producing 2-hydroxy-3-carboxy-6-oxo-7-methylocta-2,4-dienoate. The ring cleavage product is decarboxylated by 2-hydroxy-3-carboxy-6-oxo-7-methylocta-2,4-dienoate decarboxylase CmtD into 2-hydroxy-6-oxo-7-methylocta-2,4-dienoate. The genes *cmtEFHG* encode 2-hydroxy-6-oxo-7-methylocta-2,4-dienoate hydrolase, 2-hydroxypenta-2,4-dienoate hydratase, 2-oxo-4-hydroxyvalerate aldolase, and acetaldehyde dehydrogenase (acylating). These enzymes transform 2-hydroxy-6-oxo-7-methylocta-2,4-dienoate into isobutyrate, pyruvate and acetyl-CoA [5]. *B*. *xenovorans* LB400 is a model bacterium for the study of the degradation of polychlorobiphenyls (PCBs) and a wide range of aromatic compounds [12–18]. Recently, the biosynthesis of a non-ribosomal peptide hydroxamate-type siderophore in strain LB400 has been reported [19]. LB400 genome has a size of 9.73 Mbp distributed in a major chromosome (C1) (4.90 Mbp), a minor chromosome (C2) (3.36 Mbp) and a megaplasmid (MP) (1.47 Mbp) [14]. Genomic analysis of *B*. *xenovorans* strain LB400 revealed the presence of genes encoding an unusual high number of central and peripheral pathways for the degradation of aromatic compounds [14]. However, the function of genes encoding diverse aromatic catabolic routes remains to be elucidated. In the present report, the functionality of the *p*-cymene peripheral and the 2,3-dihydroxy-*p*-cumate central pathways were studied. In addition, the response of LB400 cells to *p*-cymene was characterized.

## Materials and Methods

### Chemicals

*p*-Cymene (*p*-isopropyltoluene, >98% purity) and *p*-cumate (*p*-isopropylbenzoate, 98% purity) were obtained from Sigma-Aldrich (Saint Louis, MO, USA).

### Bacterial strain and culture conditions

*B*. *xenovorans* strain LB400 was cultivated in M9 minimal medium with trace solution and glucose (5 mM), succinate (5 mM), *p*-cumate (5 mM) or *p*-cymene (vapor phase) as carbon and energy sources at 30°C [15]. *p*-Cymene was provided in vapor phase in absence or presence of succinate (5 mM). Growth was determined by measuring turbidity at 525 and 600 nm and by counting colony-forming units (CFU). Aliquots taken from bacterial cultures were diluted and plated on Luria-Bertani (LB) medium. CFU/mL values were calculated as the mean ± SD of at least three independent experiments [15,20].

### RNA isolation and RT-PCR

RNA was isolated from LB400 cells grown until mid-exponential phase (Turbidity_525nm_ = 0.3–0.5) on glucose, succinate, *p*-cumate, *p*-cymene and *p*-cymene plus succinate. RNA was isolated using the RNeasy mini kit (Qiagen, Hilden, Germany). DNase I treatment was performed using the RNase-Free DNase Set (Qiagen, Hilden, Germany). The RNA was quantified using a Qubit fluorometer (Invitrogen, Carlsbad, CA, USA). In this study, specific primers for *cymAb* (BxeA3559) and *cmtAb* (BxeA3556) genes were designed and used. For *cymAb* gene the primers cymAbf 5’-CAAAGGACGAACTGACGGTTGT-3’ and cymAbr 5’-CCGATTGCATCAAGCTGGTAGA-3’) were used. For *cmtAb* gene the primers cmtAbf 5’-AACCTGCTCGTAACGATGGGAA-3’ and cmtAbr 5’- ACCAGCCATTGACCAGCATGTA -3’, were used. Reverse transcription-PCR (RT-PCR) was carried out with 40 μg of total RNA and the sequence-specific primers using SuperScript One-step RT-PCR with Platinum Taq (Invitrogen, Carlsbad, CA, USA). Amplification of the 16S rRNA gene was performed as a control using the primers 27f (5’-AGAGTTTGATCMTGGCTCAG-3’) and 1492r (5’-TACGGYTACCTTGTTACGACTT-3’) as reported [16,17]. Negative and positive controls were included in each RT-PCR assay. The transcription of the 16S rRNA gene was used as a constitutively expressed reference gene. At least three independent RNA samples were collected at each condition and two independent RT-PCR reactions for each sample were done to assess reproducibility.

### Enzymatic activity

The *p*-cumate dioxygenase CmtA activity was measured in LB400 cells. LB400 cells grown overnight in LB medium and washed with M9 medium were resuspended until a turbidity at 600 nm of 3.0 in the same medium supplemented with 2.5 mM *p*-cumate and 0.3% lactate [5]. The mixtures were incubated with shaking at 30°C. Samples were taken immediately after the addition of cells, and at 2 h intervals for 24 h. Cells were removed by centrifugation, and the spectra of supernatants (diluted 10 fold in M9 medium) were measured. The UV-visible spectra (200–350 nm) were recorded with a Perkin-Elmer Lambda 25 spectrophotometer. The decrease of the absorbance maxima of *p*-cumate (~230 nm) through time indicates substrate oxidation [5]. Assays with boiled LB400 cells and with *E*. *coli* DH10B cells were used as negative controls. Absorbance values are the mean ± SD of at least three independent experiments.

### Bioinformatic analyses of *cym* and *cmt* genes

The *cym* and *cmt* genes and the neighborhood were analyzed in the genome of strain LB400. The genes were analyzed and depicted using the Vector NTI suite 9.0 software. For the sequence alignment the database of the National Center for Biotechnology Information (NCBI/BLAST Home) (http://blast.ncbi.nlm.nih.gov/Blast.cgi) was used.

### Electronic microscopy

LB400 cells were grown until exponential phase on succinate, *p*-cymene, *p*-cumate, *p*-cymene plus succinate as the sole carbon sources. Samples were fixed with Karnowsky fixative solution (2.5% glutaraldehyde, 3.0% formaldehyde) in 0.2 M cacodylate buffer (pH 7.4) for 2 h. Then cells were washed in the same buffer solution containing 5% sucrose. Samples were post fixed with 2% osmium tetroxide and dehydrated in ethanol and acetone series. Finally samples, were embedded in an epoxy resin (Eponate 812) and polymerized for 72 h at 60°C. Thin sections (500 nm) were obtained with diamond knife in an Ultracut E ultramicrotome (Reichert) and mounted onto 200-mesh copper grids. Sections were contrasted with uranyl acetate and lead citrate and observed with a Zeiss EM900 electron microscope [15,21,22]. The micrographs shown were representative and selected from, at least, 10 fields.

### Two-dimensional gel electrophoresis (2-DE)

2-DE gels with non-equilibrium pH gradient electrophoresis (pH 3–10) were performed as previously described with minor modifications [15,16,23]. Cell cultures were grown using *p*-cymene (vapor phase) or glucose (5 mM) as sole carbon source. Cells were harvested at exponential phase (Turbidity_525nm_ = 0.6) by centrifugation and disrupted with a sonicator. The proteins were precipitated with trichloroacetic acid overnight at 4°C. The precipitate was harvested by centrifugation and washed 2 times with acetone and dried thoroughly. Dried samples were suspended in lysis buffer (9.5 M urea, 2% v/v IGEPAL CA-630, 2% ampholytes (1.6% ampholytes pH 5–7, 0.4% ampholytes pH 3–10, Bio-Rad) and 5% *β*-mercaptoethanol) [16,24] to a final protein concentration of 230 mg/mL. Protein concentration was measured using the Quantit-iT Protein Assay kit (Invitrogen, Carlsbad, CA, USA) [25]. NEPHGE gels were electrophoresed for 8.5 h at 400 V. The second dimension was performed in 11% polyacrilamide-SDS gels. Proteins were visualized with Coomassie brilliant blue G-250. To visualize proteins, the gels were stained with colloidal Coomassie and analyzed with ImageMaster 2-D gel analysis 7.0 software (General Electric Healthcare Bio-Sciences, Pittsburgh, USA). Three biological replicates were analyzed by 2-DE gels to increase the external reproducibility and at least two gels of each assay were analyzed. The images of the gels were captured using the Gel-doc system and Quantity One software (BioRad). The raw images were analyzed using the ImageMaster 2-D gel analysis version 7.0. At least six gels of each condition (glucose versus *p*-cymene as sole carbon source) were compared and analyzed. To standardize the quantities of the protein expressed, the ratio of the density of the protein (intensity/mm^2^) to a reference protein was calculated. Only significant changes in spot intensity were reported (≥1.5-fold).

### Protein identification by mass spectrometry

In order to identify proteins the spots were recovered from the 2-DE gels and prepared as described [15,16]. The digestion of proteins was performed with sequencing-grade trypsin (2 μg/mL). Peptide extracts were eluted directly from the ZipTip microcolumns with saturated alpha-cyano-4-hydroxycinnamic acid directly onto the MALDI target. For matrix-assisted laser desorption ionization-time of flight (MALDI-TOF) analysis, the peptide extracts were analyzed with a MALDI-TOF Ultraflex apparatus (Bruker, Bremen, Germany). The peptide fingerprints obtained by MALDI-TOF mass spectrometry were used for searches in the NCBI protein database with the Matrix science MASCOT search tool. The complete sequences of the proteins were obtained and BLAST searches were executed with the FASTA tool for the identification and similarity data analyses.

### Biofilm quantification

Biofilm formation was determined as the capability of *B*. *xenovorans* LB400 to adhere to the walls and base of polycarbonate microtiter plates. *B*. *xenovorans* LB400 were grown on M9 medium with glucose as carbon source until exponential phase (6.5 x 10^7^ CFU/mL; Turbidity_600 nm_ = 0.6) and then cultivated in cell culture plates (polycarbonate) with *p*-cumate (5 mM) or *p*-cymene (vapor phase) without shaking (adhesion phase) at 30°C. *p*-Cymene was provided in vapor phase as the compound has very low aqueous solubility. Biofilm formation was allowed to proceed for 24, 48 and 72 h at 30°C. The assays were carried out on three different replicates in triplicate.

### Confocal laser scanning microscopy (CLSM)

After LB400 biofilm formation, the planktonic cells were removed and gently rinsed in phosphate-buffered saline (PBS) for 15 seconds to not disrupt the biofilm. The biofilms were stained using the Molecular Probes’ Live/Dead BacLight Bacterial Viability kit, which is composed of SYTO-9 and propidium iodide (PI) (Molecular Probes, Eugene, USA). SYTO-9 is a green-fluorescent nucleic acid stain, generally labeling live and dead microorganisms. PI is a red fluorescent nucleic acid stain that penetrates only the cells with damaged membranes, thus visualizing dead microbes. Observations were made at a 63× magnification using a Leica TCS-SP2-AOBS-UV CLSM equipped with an argon ion laser. Images were analyzed using LCS software from Leica. Projections were obtained in the planes x–y (individual scans at 0.5 μm intervals) and x–z (images at 6 μm intervals). In addition, the biofilm viability was determined by CFU counting. Aliquots taken from bacterial biofilm were diluted and plated on LB medium. CFU/mL values were calculated as the mean ± SD of at least three independent experiments [15,20].

## Results

### Genomic analysis of the *p*-cymene and 2,3-dihydroxy-*p*-cumate catabolic pathways genes

**Fig 1:**
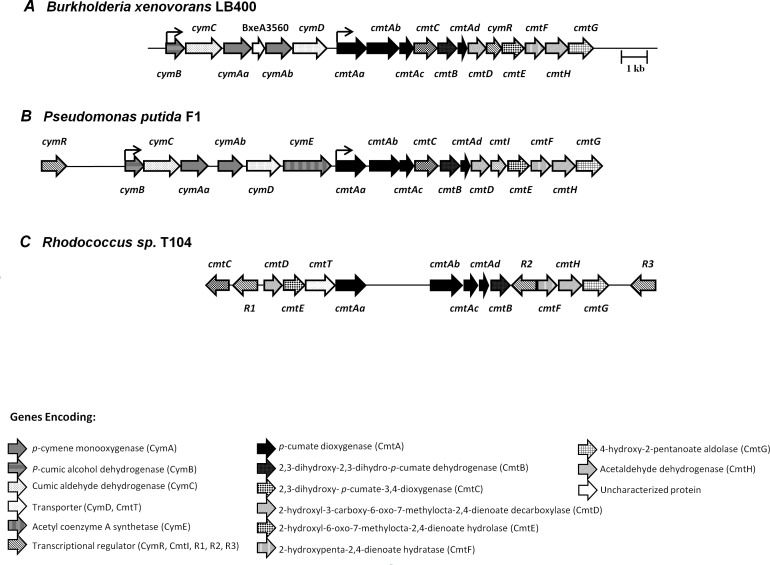
Organization of the genes encoding the *p*-cymene peripheral and the 2,3-dihydroxy-*p*-cumate central pathways in *B*. *xenovorans* LB400 and comparison with related bacterial gene clusters. Organization of genes encoding the *p*-cymene, *p*-cumate and the 2,3-dihydroxy-*p*-cumate pathways in *Burkholderia xenovorans* LB400 (A), *Pseudomonas putida* F1 (B) and *Rhodococcus sp*. T104 (C). The *cym* genes encode proteins from the *p*-cymene catabolic pathway, and the *cmt* genes encode proteins from the *p*-cumate and 2,3-dihydroxy-*p*-cumate catabolic pathways.

The genes encoding the *p*-cymene and 2,3-dihydroxy-*p*-cumate pathways from strain LB400 were studied. The *cym* genes that encode proteins of the *p*-cymene catabolic pathway are located in the *cymBCAaAbD* gene cluster at C1 (Fig 1). The predicted *cym* genes from *B*. *xenovorans* LB400 are listed in Table 1.

**Table 1:** Predicted genes encoding the *p*-cymene peripheral and the 2,3-dihydroxy-*p*-cumate central pathways from *B*. *xenovorans* LB400.

Gene	ORF	aa	Related gene products		
			Protein	Function (Organism)	% Id (aa)
*cymAa*	BxeA3561	375	CymAa	*p*-cymene monooxygenase hydroxylase subunit (*P*. *putida* F1)	77 (376)
*cymAb*	BxeA3559	349	CymAb	*p*-cymene monooxygenase reductase subunit (*P*. *putida* F1)	77 (349)
*cymB*	BxeA3563	252	CymB	*p*-cumic alcohol dehydrogenase (*P*. *putida* F1)	83 (252)
*cymC*	BxeA3562	494	CymC	*p*-cumic aldehyde dehydrogenase (*P*. *putida* F1)	87 (494)
*cymD*	BxeA3558	460	CymD	Outer membrane protein / *p*-cymene transporter (*P*. *putida* F1)	60 (460)
*cymR*	BxeA3550	209	CymR	TetR family transcriptional regulator (*P*. *putida* F1)	71 (203)
*cmtAa*	BxeA3557	402	CmtAa	*p*-cumate dioxygenase ferredoxin reductase subunit (*P*. *putida* F1)	69 (402)
*cmtAb*	BxeA3556	434	CmtAb	*p*-cumate dioxygenase large subunit of the terminal dioxygenase (*P*. *putida* F1)	82 (434)
*cmtAc*	BxeA3555	180	CmtAc	*p*-cumate dioxygenase small subunit of the terminal dioxygenase (*P*. *putida* F1)	82 (180)
*cmtAd*	BxeA3552	116	CmtAd	*p*-cumate dioxygenase ferredoxin subunit (*P*. *putida* F1)	73 (118)
*cmtB*	BxeA3553	256	CmtB	2,3-dihydroxy-2,3-dihydro-*p*-cumate dehydrogenase (*P*. *putida* F1)	78 (259)
*cmtC*	BxeA3554	314	CmtC	2,3-dihydroxy-*p*-cumate-3,4-dioxygenase (*P*. *putida* F1)	83 (312)
*cmtD*	BxeA3551	244	CmtD	HCOMODA decarboxylase (*P*. *putida* F1)	76 (243)
*cmtE*	BxeA3549	301	CmtE	HOMODA hydrolase (*P*. *putida* CE2010)	83 (259)
*cmtF*	BxeA3548	262	CmtF	2-hydroxypenta-2,4-dienoate hydratase (*P*. *putida* F1)	83 (262)
*cmtG*	BxeA3546	340	CmtG	4-hydroxy-2-oxovalerate aldolase (*P*. *putida* F1)	81 (350)
*cmtH*	BxeA3547	314	CmtH	Acetaldehyde dehydrogenase (*P*. *putida* F1)	89 (314)

**Fig 2:**
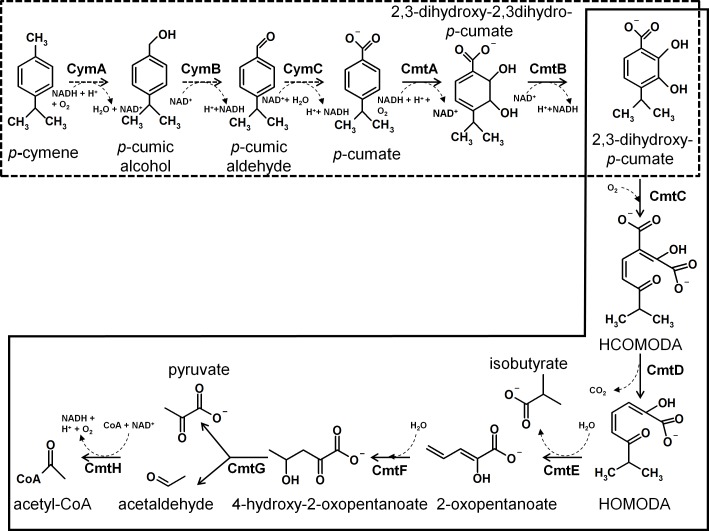
Model of *p*-cymene and 2,3-dihydroxy-*p*-cumate catabolic pathways in *B*. *xenovorans* LB400. The box with dotted border indicates the *p*-cymene peripheral pathway, which converts *p*-cymene into 2,3-dihydroxy-*p*-cumate. The substrate is *p*-cymene and the products are *p*-cumic alcohol, *p*-cumic aldehyde, *p*-cumate, 2,3-dihydroxy-2,3dihydro-*p*-cumate and 2,3-dihydroxy-*p*-cumate. The enzymes are CymA (*p*-cymene monooxygenase), CymB (*p*-cumic alcohol dehydrogenase) CymC (*p*-cumic aldehyde dehydrogenase), CmtA (*p*-cumate dioxygenase) and CmtB (2,3-dihydroxy-2,3-dihydro-*p*-cumate dehydrogenase). The reactions of the 2,3-dihydroxy-*p*-cumate central pathway are presented in a box with continuous border. The substrate is 2,3-dihydroxy-*p*-cumate and the final products are isobutyrate, pyruvate and acetyl-CoA. The enzymes are CmtC (2,3-dihydroxy-*p*-cumate-3,4-dioxygenase), CmtD (2-hydroxy-3-carboxy-6-oxo-7-methylocta-2,4-dienoate decarboxylase), CmtE (2-hydroxy-6-oxo-7-methylocta-2,4-dienoate hydrolase), CmtF (2-hydroxypenta-2,4-dienoate hydratase), CmtG (4-hydroxy-2-pentanoate aldolase) and CmtH (acetaldehyde dehydrogenase).

The *cymAa* (BxeA3561) and *cymAb* (BxeA3559) genes encode for the hydroxylase and reductase subunits of the *p*-cymene monooxygenase. The *cymAaAb* gene products from strain LB400 showed 77% (376/349) aa identity with the *p*-cymene monooxygenase CymA proteins from *P*. *chlororaphis subsp*. *aureofaciens* and *P*. *putida* F1. The *cymB* (BxeA3563) and *cymC* (BxeA3562) genes encode the *p*-cumic alcohol dehydrogenase and the *p*-cumic aldehyde dehydrogenase, respectively, showing 83% and 87% identity with the *cymB* and *cymC* gene products from *P*. *putida* F1. The *cymD* gene (BxeA3558) encodes an outer membrane protein that possesses 60% identity with the *p*-cymene CymD transporter from *P*. *putida* F1. The *cmtAaAbAcCBAdDEFG* gene cluster from strain LB400 encodes the enzymes for the *p*-cumate degradation via the 2,3-dihydroxy-*p*-cumate central catabolic pathway (Fig 2). The *cmt* gene cluster is located at C1 next and downstream from the *cym* gene cluster. The characterization of the *cmt* genes from *B*. *xenovorans* LB400 are shown in Table 1. The *cmtAa* (BxeA3557), *cmtAb* (BxeA3556), *cmtAc* (BxeA3555) and *cmtAd* (BxeA3552) genes encode the ferredoxin reductase subunit component, large and small subunits of the terminal dioxygenase and ferredoxin component of the *p*-cumate dioxygenase, which is a type I extradiol dioxygenase. CmtA catalyzes the oxidation of *p*-cumate into *cis*-2,3-dihydroxy-2,3-dihydro-*p*-cumate. The *cmtAa*, *cmtAb*, *cmtAc* and *cmtAd* gene products showed ≥ 69% identity with CmtAa, CmtAb, CmtAc and CmtAd proteins from *P*. *putida* F1 [5]. The *cmtB* (BxeA3553) and *cmtC* (BxeA3554) genes encode the 2,3-dihydroxy-2,3dihydro-*p*-cumate dehydrogenase and the 2,3-dihydroxy-*p*-cumate-3,4-dioxygenase, respectively. The CmtB catalyzes the conversion of *cis*-2,3-dihydroxy-2,3-dihydro-*p*-cumate into 2,3-dihydroxy-*p*-cumate. CmtC transforms 2,3-dihydroxy-*p*-cumate into 2-hydroxy-3-carboxy-6-oxo-7-methylocta-2,4-dienoate (HCOMODA). The *cmtD* and *cmtE* gene products showed 76% and 83% identity with CmtD y CmtE proteins from *P*. *putida* F1 [5]. The *cmtD* (BxeA3551) and *cmtE* (BxeA3549) genes encode the 2-hydroxy-3-carboxy-6-oxo-7-methylocta-2,4-dienoate decarboxylase (HCOMODA decarboxylase) and the 2-hydroxy-6-oxo-7-methylocta-2,4-dienoate hydrolase (HOMODA hydrolase), respectively (Fig 2). The *cmtF* (BxeA3548), *cmtG* (BxeA3547) and *cmtH* (Bxe3546) genes encode the 2-hydroxypenta-2,4-dienoate hydratase, the 4-hydroxy-2-oxovalerate aldolase and the acetaldehyde dehydrogenase, respectively. The last metabolic steps catalyze the conversion of 2-hydroxypenta-2,4-dienoate into pyruvate and acetyl-CoA (Fig 2). The BxeA3550 gene located between *cmtD* and *cmtE* genes from strains LB400 showed 78% identity with the transcriptional regulator CymR from *P*. *putida* F1 [6] (Table 1). The CymR protein is a TetR-type transcriptional regulator. In *P*. *putida* F1 the CymR protein binding sequence 5`-ACAAACAGAC-N6-GTCTGTTTGT-3` is located upstream of the *cmtAa* gene [26]. The *in silico* analysis of LB400 genome, showed TetR-type binding sequences upstream of the *cymB* gene (5´-ACAAACAGAC-N6-GTCTGTTTGT-3´) and upstream of the *cmtAa* gene (5´-ATAAACAGAC-N6-GTCGGTTTGT-3´).

The genetic organization of the *cym* and *cmt* gene clusters in *B*. *xenovorans* LB400 is similar to the organization of *cym* and *cmt* operons in *P*. *putida* F1 (Fig 1), except for the position of the *cymR* gene and the absence in strain LB400 of the *cymE* and *cmtI* (subsequently renamed *cmtR)* genes. Strain LB400 has a *cymR* regulator gene located in the position of the *cmtI* gene from *P*. *putida* F1. *Rhodococcus* sp. T104 showed a different *cmt* genes organization than *B*. *xenovorans* strain LB400 and *P*. *putida* F1 (Fig 1). Strain T104 is able to degrade *p*-cumate, other terpenoids such as limonene and pinene, and other compounds such as biphenyl and abietic acid [9]. The main differences in T104 gene cluster are the presence of three IclR-type transcriptional regulators genes flanking the *cmt* gene cluster, and the *cmtT* gene that may encode a transporter for the import of aromatic compounds. Interestingly, the organization of *cym* genes in the strain LB400 showed the absence of the *cymE* gene and the presence of the BxeA3560 gene in the *cym* gene cluster. The function of BxeA3560 gene in the *cym* cluster in the *p*-cymene/*p*-cumate metabolism is unknown.

### Functionality of *p*-cymene and *p*-cumate catabolic pathways

**Fig 3:**
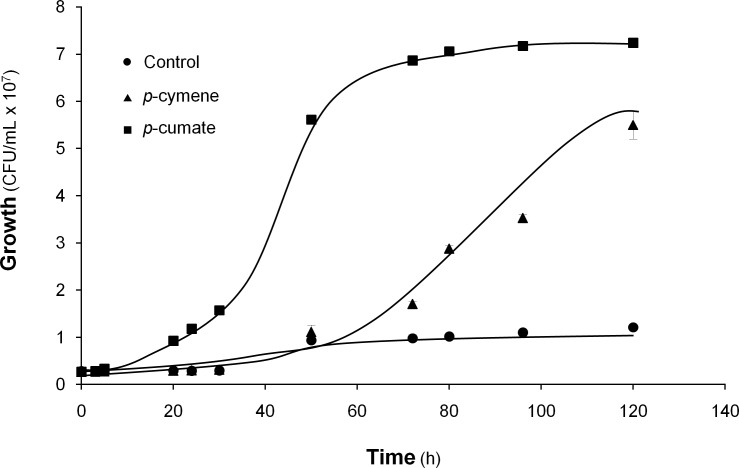
Growth of *B*. *xenovorans* LB400 on *p*-cymene and *p*-cumate. LB400 cells were grown in M9 medium using *p*-cymene (vapor phase) or *p*-cumate (5 mM) as the sole carbon and energy source. Cells without carbon source were used as control. CFU/mL values were calculated as the mean ± SD of at least three independent experiments.

To investigate if the *p*-cymene and *p*-cumate catabolic pathways are functional in strain LB400, in a first approach growth on *p*-cymene and *p*-cumate was studied. As shown in Fig 3, *B*. *xenovorans* LB400 was able to grow on *p*-cymene as the sole carbon and energy source. Strain LB400 was also able to grow on *p*-cumate (Fig 3). *B*. *xenovorans* LB400 attained higher cell concentration at stationary phase growing on *p*-cumate (7.0 × 10^7^ CFU/mL) that on *p*-cymene (4.5 × 10^7^ CFU/mL). LB400 cells grown on *p*-cymene showed a slower growth compared to the cells grown on the *p*-cumate. *p*-Cumate is more hydrophilic and, thus, a higher concentration in the aqueous phase is expected, indicating higher substrate availability for bacterial growth. Cells growth on *p*-cymene and *p*-cumate as sole carbon and energy sources indicates functional *p*-cymene and *p*-cumate catabolic pathways in *B*. *xenovorans* LB400.

### Transcriptional analyses of key *cym* and *cmt* genes and *p*-cumate dioxygenase activity assay

**Fig 4:**
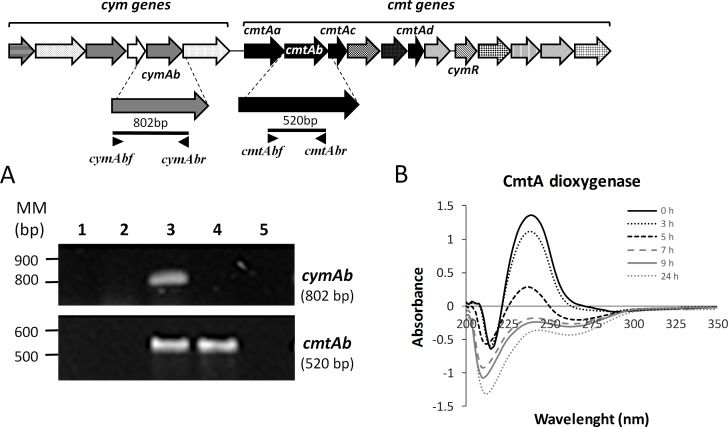
Expression of the *cymAb* and *cmtAb* genes during LB400 growth on *p*-cymene and *p*-cumate, and activity of *p*-cumate dioxygenase. (A) Expression of *cymAb* and *cmtAb* genes during LB400 growth on *p*-cymene and *p*-cumate. Expression of *cym*Ab (BxeA3559) and *cmt*Ab (BxeA3556) genes in LB400 cells grown in glucose (lane 1), succinate (lane 2), *p*-cymene (lane 3), *p*-cumate (lane 4) and *p*-cymene plus succinate (lane 5). RT-PCR assays were performed using RNA from LB400 cells collected at exponential growth phase. The expression of 16S rRNA was used as a control to normalize across samples. At least three independent RNA samples were collected at each condition and two independent RT-PCR reactions for each sample were done to assess reproducibility. (B) Activity of the *p*-cumate dioxygenase CmtA in LB400 cells. LB400 cells grown overnight in LB medium and washed with M9 medium were incubated with 2.5 mM *p*-cumate and 0.3% lactate. Absorbance values are the mean ± SD of at least three independent experiments.

To study the expression of the genes encoding *p*-cymene and *p*-cumate catabolic pathways in strain LB400, transcriptional analysis of key catabolic genes was performed. Cells grown in *p*-cymene, *p*-cumate, glucose and succinate were analyzed by RT-PCR. The expression of the *cymAb* gene that encodes the *p*-cymene monooxygenase reductase subunit from strain LB400 was analyzed. The *cymAb* gene was only transcribed during LB400 growth on *p*-cymene (Fig 4A), but not during growth on *p*-cumate, succinate or glucose. These results indicate the specific induction the expression of the LB400 *cymAb* gene by *p*-cymene. The transcription of the *cmtAb* gene that encodes the key *p*-cumate dioxygenase large subunit during growth of strain LB400 on different substrates was studied. The *cmtAb* gene was expressed during growth of strain LB400 on *p*-cymene as well as during growth on *p*-cumate (Fig 4A). In contrast, *cmtAb* transcripts were not observed during growth of strain LB400 on glucose, succinate or *p*-cymene plus succinate. These results suggest that the transcription of the *cmtAb* gene is induced by *p*-cumate and probably by *p*-cymene. In addition, these results suggest that succinate repressed the expression of the *cmtAb* gene. The enzymatic assay shows that LB400 *p*-cumate dioxygenase oxidized *p*-cumate (Fig 4B), indicating that this key enzyme is active.

### Effects of *p*-cymene and *p*-cumate on cell morphology

**Fig 5:**
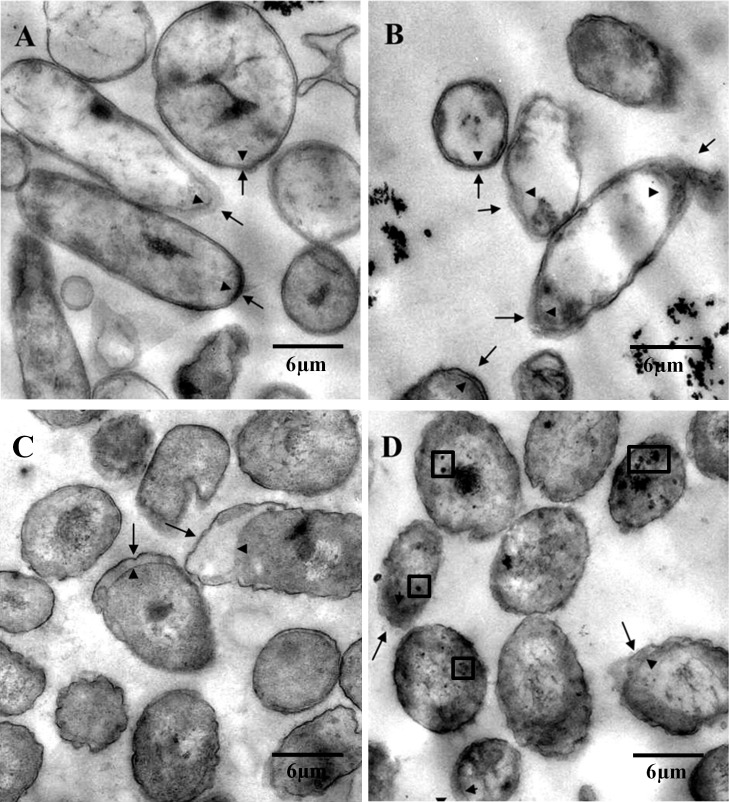
Effects of growth on *p*-cymene and *p*-cumate on the cell morphology of *B*. *xenovorans* LB400. Cells were grown in M9 minimal medium using succinate (A), *p*-cumate (B), *p*-cymene (C), and succinate plus *p*-cymene (D) as sole carbon and energy sources. Arrows and arrowheads indicate the outer and the inner membrane, respectively. Some internal granules are indicated within boxes. The micrographs shown were representative and selected from, at least, 10 fields.

The effects of the growth on *p*-cymene or *p*-cumate on *B*. *xenovorans* LB400 cell morphology were studied by transmission electron microscopy. Micrographs of LB400 cells grown in *p*-cymene or *p*-cumate as sole carbon source are shown in Fig 5. *B*. *xenovorans* LB400 grown in *p*-cymene or *p*-cumate exhibited fuzzy outer membrane and a wider periplasmic space towards the cell poles. However, strain LB400 grown on *p*-cymene and succinate showed a less fuzzy outer membrane, and some electron-dense granules in the cytoplasm (Fig 5D). Micrographs of *p*-cumate-grown cells showed cell lysis and cell debris. Cell morphology analysis showed a marked effect of *p*-cymene at the cell membranes. Therefore, the effect of growth on the *p*-cymene on the proteome of strain LB400 was studied.

### Effect of *p*-cymene on LB400 proteome and biofilm formation

**Fig 6:**
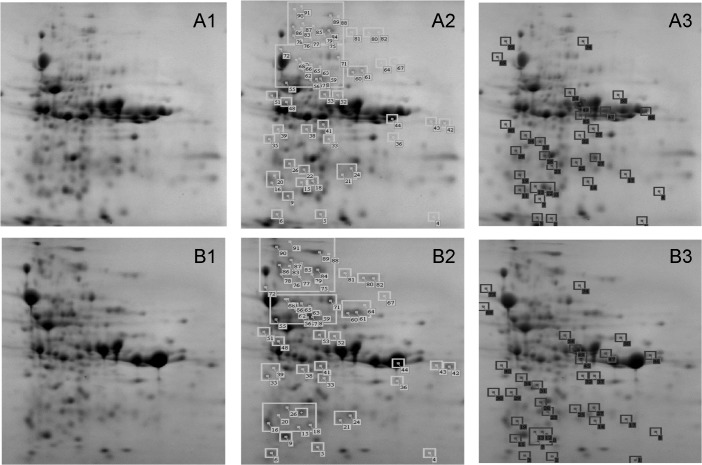
Effects of *p*-cymene on the proteome of *B*. *xenovorans* LB400. Total protein pattern of LB400 cells grown in M9 medium using glucose (A1) and *p*-cymene (B1) as sole carbon and energy sources. Induced proteins in *p*-cymene-grown cells on the total protein pattern of strain LB400 grown in glucose (A2) and *p*-cymene (B2) are boxed. Proteins repressed in *p*-cymene-grown cells on the total protein pattern of strain LB400 grown in glucose (A3) and *p*-cymene (B3) are boxed. The gels depicted are representative of at least three biological replicates and at least six 2-DE gels.

The effect of *p*-cymene on LB400 proteome was studied by 2-DE with a nonlinear pH gradient from 3 to 10. Protein pattern of *p*-cymene-grown cells and glucose-grown cells were compared. The levels of approximately 150 proteins changed in LB400 *p*-cymene-grown cells compared with glucose-grown cells. Fig 6 illustrates representative LB400 protein patterns of cells grown on glucose and *p*-cymene. 2-DE analyses revealed that in *p*-cymene-grown cells, 59 proteins were up regulated compared to glucose grown cells, whereas 32 proteins were down regulated (Fig 6B). 28 proteins were only observed in the protein pattern of LB400 cells grown on glucose, whereas 31 proteins were only visualized in *p*-cymene-grown cells. Proteins 90, 83, 81, 78, 66 and 36 are highly induced (≥ 5 fold) by *p*-cymene. In LB400 cells grown in *p*-cymene, proteins 3 and 10 were strongly repressed (~8 fold), whereas proteins 1, 11, 12, 14 and 40 were also highly repressed (4 fold). Peptide fingerprints from proteins were obtained by MALDI-TOF mass spectrometry and proteins were identified using NCBI protein database with the Matrix science MASCOT search tool. The proteins from strain LB400 induced and repressed during growth on *p*-cymene are shown in the Table 2 and Table 3.

**Table 2:** Identification of LB400 proteins induced during growth on *p*-cymene.

Spot No ^a^	Gene	pI/M_r_ (kDa)^b^	Similarity (Organism)	Id (%)	Fold Change ^c^
**Stress response proteins**					
5	BxeB2843	6.05 / 14.32	Organic hydroperoxide reductase (*Pseudomonas syringae pv*. *oryzae* str. 16)	71	1.7062
16	BxeB1205	5.10 / 20.75	Alkyl hydroperoxide reductase (AhpC) (*Tolumonas auensis* DSM9187)	82	1.6223
52	BxeB2294	7.38 / 47.40	CopA Multicopper oxidase type 3 (*Ralstonia pickettii* 12J)	78	2.2005
68	BxeC0135	5.05 / 57.36	Chaperonine GroEL (*Burkholderia vietnamiensis*)	97	2.5935
77	BxeB0192	5.57 / 62.18	DnaK (*Burkholderia mallei* ATCC)	92	4.7202
87	BxeA2374	5.52 / 96.06	ATP-dependent Clp protease ATP-binding subunit ClpB (*Cupriavidus taiwanensis* LMG 19424)	82	2.8469
**Transport and membrane proteins**					
9	BxeA3921	6.51 / 18.64	OmpA/motility protein B (MotB) (*Cupriavidus metallidurans* CH34)	73	1.5358
15	BxeB2786	9.15 / 39.51	OmpC family outer membrane protein, porin (*Burkholderia phytofirmans* PsJN)	97	1.6974
33	BxeA3631	8.76 / 25.34	Exported protein (*Ralstonia solanacearum* UW551)	50	3.2278
36	BxeA1997	9.30 / 34.18	Ribose ABC transporter binding protein component (*Burkholderia pseudomallei* MSHR346)	87	5.6487
43	BxeC1181	9.30 / 37.28	ABC transporter periplasmic protein (*Halomonas elongata* DSM 2581)	47	2.2566
44	BxeA3558	8.73 / 48.70	Outer membrane protein CymD (*Pseudomonas putida* F1)	60	3.2757
57	BxeB2189	6.84 / 47.38	Lysine/arginine/ornithine ABC transporter (*Chromobacterium violaceum* ATCC 12472)	75	2.1843
61	BxeA0182	8.66 / 60.20	ABC transporter, substrate-binding protein (*Pseudomonas putida* KT2440)	66	2.1089
100	BxeA1941	7.81 / 39.41	Outer membrane lipoprotein (*Cupriavidus taiwanensis* LMG 19424)	57	Cym
106	BxeA0720	8.78 / 47.72	ABC sugar transporter sorbitol-binding protein / mannitol-binding protein (*Ralstonia solanacearum* GMI1000)	82	Cym
**Aromatic metabolism**					
22	BxeC1196	5.72 / 24.99	BphA2 (biphenyl 2,3-dioxygenase beta subunit) (*Pseudomonas* sp. B4)	100	3.3276
**Synthesis of proteins and RNA**					
39	BxeA0299	5.40 / 43.06	Elongation factor Tu (*Cupriavidus taiwanensis* LMG 19424)	94	2.4472
72	BxeA0982	5.05 / 63.03	30S ribosomal protein S1 (*Ralstonia eutropha* H16)	84	3.1461
86	BxeA0311	5.24 / 77.53	Elongation factor G (*Ralstonia eutropha* H16)	87	1.7850
101	BxeA0340	5.64 / 35.66	DNA-directed RNA polymerase alpha subunit (*Ralstonia eutropha* JMP134)	91	Cym
**Energy metabolism**					
48	BxeA0674	5.17 / 41.23	Succinyl-CoA synthetase beta subunit (*Cupriavidus metallidurans* CH34)	84	1.6365
89	BxeB2903	6.14 / 98.41	Aconitate hydratase AcnA (*Ralstonia solanacearum* UW551)	83	2.2703
91	BxeB1533	5.44 / 92.86	Bifunctional aconitate hydratase 2 /2-methylisocitrate dehydratase (*Burkholderia phymatum* STM815)	97	2.0201
55	BxeA0040	5.24 / 50.46	ATP synthase beta subunit (*Cupriavidus taiwanensis* LMG 19424)	87	1.8236
51	BxeA1573	4.80 / 45.58	Enolase (*Ralstonia solanacearum*)	91	1.5069
56	BxeA0690	5.81 / 51.27	Pyruvate kinase (*Cupriavidus taiwanensis* LMG 19424)	76	1.7239
**Other functions**					
6	BxeA4379	5.23 / 16.1	Transcriptional regulators TraR (*Cupriavidus necator* JMP134)	87	1.5253
26	BxeA4345	5.45 / 28.93	Ferredoxin-NADP(+) reductase (*Burkholderia thailandesis* E264)	74	1.5246
35	BxeA0263	5.58 / 23.52	Conserved exported protein of unknown function (*Ralstonia solanacearum* PSI07)	56	1.6975
38	BxeB0704	5.96 / 31.88	Oxidoreductase 3-hydroxyisobutyrate dehydrogenase (*Bradyrhizobium* sp. ORS 278)	66	2.1293
41	BxeA4132	6.07 / 32.02	4-diphosphocytidyl-2-methyl-D-erythritol kinase (*Ralstonia solanacerum* CFBP2957)	57	2.3570
53	BxeA3332	6.14 / 43.97	Class I and II aminotransferase (*Ralstonia eutropha* JMP134)	67	1.6558
58	BxeA0708	9.16 / 42.37	Membrane fusion protein (*Pseudomonas syringae* pv. *tabaci* ATCC 11528)	67	1.6669
60	BxeA1706	6.62 / 52.02	Inosine 5'-monophosphate dehydrogenase (*Cupriavidus taiwanensis* LMG 19424) Nucleotide biosynthesis	80	2.3189
71	BxeA4182	8.34 / 57.39	C-terminal processing peptidase-3, periplasmic (*Ralstonia eutropha* H16)	69	1.6748
76	BxeA1543	5.37 / 63.21	Dihydrolipoamide dehydrogenase (*Ralstonia pickettii* 12D)	74	4.3426
90	BxeB1939	5.67 / 47.56	Hypothetical protein Bpro_4171 (*Polaromonas* sp. JS666)	51	11.9506

^a^ Spot number shown in Fig 5.

^b^ Theoretical isoelectric point and molecular mass.

^c^ Change in expression of the protein. Positive values indicate induction of protein during growth on *p*-cymene compared to glucose-grown cells Cym, only detected in *p*-cymene-grown cells.

**Table 3:** Identification of LB400 proteins repressed during growth on *p*-cymene.

Spot No.^a^	Gene	pI/M_r_ (kDa)^b^	Similarity (Organism)	Id (%)	Fold Change ^c^
**Stress response proteins**					
124	BxeA3816	5.80 / 10.46	Co-chaperonine GroES (*Ralstonia pickettii* 12D)	91	Glu
**Transport and membrane proteins**					
10	BxeA3152	6.29 / 19.70	Fe2+ transport protein (*Achromobacter xylosoxidan*s AXX-A)	67	-10.6354
17	BxeA0391	9.48 / 24.14	ABC transporter, periplasmic ligand binding protein, toluene tolerance (*Cupriavidus metallidurans* CH34)	55	-3.5158
27	BxeB2189	8.61 / 28.23	Lysine/arginine/ornithine ABC transporter (*Chromobacterium violaceum* ATCC 12472)	75	-2.1912
37	BxeA0546	8.73 / 35.06	Monosaccharide-transporting ATPase (*Ralstonia pickettii* 12J)	69	-2.3706
49	BxeA0475	8.83 / 40.22	Porin (*Ralstonia eutropha* H16)	58	-3.3821
47	BxeA0050	8.82 / 40.39	ABC transporter periplasmic protein (*Ralstonia eutropha* H16)	71	-2.0697
129	BxeA4348	9.34 / 48.81	Amino acid permease-associated protein (*Ralstonia pickettii* 12D)	77	Glu
136	BxeA0597	8.95 / 32.51	ABC transporter periplasmic protein (*Ralstonia eutropha* H16)	64	Glu
140	BxeA3451	8.77 / 44.13	Sugar ABC transporter substrate-binding protein (*Burkholderia phytofirmans* PsJN)	72	Glu
**Aromatic metabolism**					
23	BxeB2882	5.85 / 24.78	N-(5'-phosphoribosyl)anthranilate isomerase (*Ralstonia solanacearum* UW551), biosynthesis tryptophan	65	-3.5449
34	BxeC1192	5.28 / 28.90	cis-2,3-dihydroxy-2,3-dihydrobiphenyl dehydrogenase BphB (*Pseudomonas* sp. Cam-1)	99	-3.3409
**Synthesis of proteins**					
127	BxeA1009	10.52 / 14.20	50S ribosomal protein L19 (*Ralstonia pickettii* 12D)	85	Glu
**Energy metabolism**					
19	BxeA3400	5.30 / 19.24	Inorganic pyrophosphatase (*Cupriavidus metallidurans* CH34)	82	-2.6932
132	BxeA0590	6.20 / 22.18	Bifunctional 4-hydroxy-2-oxoglutarate aldolase/2-dehydro-3-deoxyphosphogluconate aldolase (*Acinetobacter* sp. ADP1)	84	Glu
138	BxeA0566	6.60 / 36.20	Glyceraldehyde 3-phosphate dehydrogenase (*Ralstonia eutropha* H16)	85	Glu
**Second messenger synthesis**					
29	BxeB2035	5.71 / 85.67	Diguanylate cyclase/phosphodiesterase (*Allochromatium vinosum* DSM 180)	37	-1.7790
**PHA metabolism and fatty acid *β*-oxidation**					
12	BxeA1544	6.18 / 20.09	Phasin (PHA-granule associated protein) (*Cupriavidus metallidurans* CH34), PhaP1	74	-4.9593
28	BxeA0762	5.93 / 26.27	3-hydroxyacyl-CoA dehydrogenase (*Ralstonia solanacearum* GMI1000)	79	-3.8809
**Other functions**					
11	BxeA4352	5.36 / 84.88	Xanthine dehydrogenase, molybdenum binding subunit apoprotein (*Ralstonia eutropha* JMP134), purine metabolism	83	-4.2960
14	BxeB1750	9.19 / 21.61	Hypothetical protein [*Pseudomonas protegens* Pf-5]	58	-4.7280
31	BxeA3631	8.76 / 25.34	Periplasmic or secreted protein (*Cupriavidus metallidurans* CH34)	56	-1.7848
135	BxeA4083	6.33 / 28.38	GntR family transcriptional regulator (*Bordetella petrii* DSM 12804)	53	Glu

^a^ Spot number shown in Fig 5.

^b^ Theoretical isoelectric point and molecular mass.

^c^ Change in expression of the protein. Negative values refer to proteins repressed during growth on *p*-cymene as the sole carbon source, compared to glucose-grown cells Glu, only detected in glucose-grown cells.

**Fig 7:**
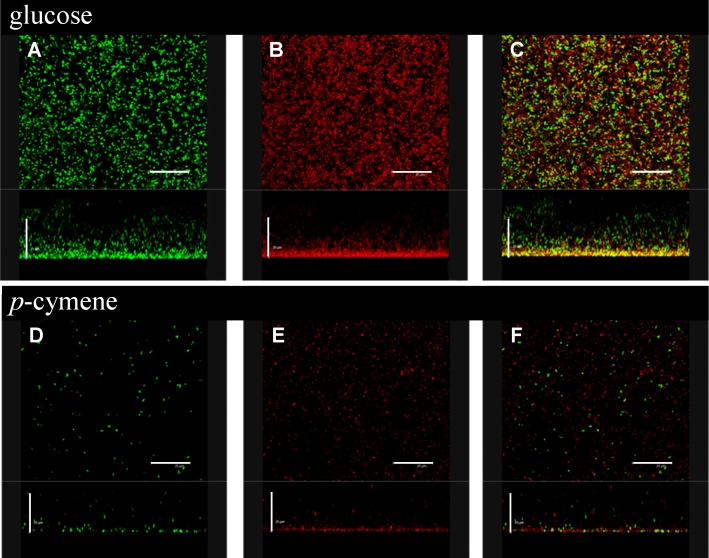
Effect of *p*-cymene on biofilm formation by *B*. *xenovorans* LB400. Cells were grown in M9 medium using glucose or *p*-cymene as sole carbon source until exponential phase (Turbidity_600nm_ = 0.6) and washed were resuspended in M9 medium supplemented with glucose (5 mM) or p-cymene (vapor phase). Microscopic characterization of *B*. *xenovorans* LB400 biofilms grown in M9 medium supplemented with glucose (5 mM) or *p*-cymene (vapor phase) during 48 h. Cells in the biofilms were stained with the BacLight kit showing viable (green fluorescence) and non-viable (red fluorescence) bacteria (C and F). The viable and non-viable cells were marked with green fluorescence (A and D). The dead cells were stained red (B and E). Images were horizontal and vertical three-dimensional reconstructed in the x–y plane and x–y plane, respectively. In all images the scale bar is 25 μm.

Five *p*-cymene-induced proteins are involved in the control of one of the cellular programs that activate bacteria in stress response (Table 2). Protein 77 (4.7 fold induced during growth in *p*-cymene) was identified as DnaK (BxeB0192). Proteins 68 and 87 (> 2 fold induced) were identified as chaperonine GroEL (BxeC0135) and chaperone ClpB (BxeA2374). Proteins 5 and 16 were identified as organic hydroperoxide reductase Ohr (BxeB2843) and alkyl hydroperoxide reductase AhpC (BxeB1205) that are involved in the detoxification of hydroperoxides. The strongly induction of molecular chaperone proteins and hydroperoxide detoxification proteins, suggest that growth on *p*-cymene constitutes a stressful condition for strain LB400. A number of the transporters and membrane proteins were induced by *p*-cymene metabolism and are listed in the Table 2. Protein 44 that showed ≥ 3 fold induction in *p*-cymene grown cells was identified as the outer membrane protein CymD (60% identity with *p*-cymene transporter CymD from *P*. *putida* F1). The *cymD* gene is located between *cymAb* (BxeA3559) and *cmtAa* (BxeA3557) at the *cym*/*cmt* gene cluster from strain LB400. Protein 36 (BxeA1997) was induced 5.6 fold and showed 87% identity to a ribose binding subunit of an ABC-type transporter system from *B*. *pseudomallei* MSHR346. Proteins 100 and 106 were only observed during growth on *p*-cymene and are an outer membrane protein and the sorbitol-binding protein of an ABC sugar transporter, respectively. Another subgroup of the *p*-cymene-induced proteins are associated to glycolysis, TCA cycle and oxidative phosphorylation. Three proteins (induced ≥1.6 times) were identified as the succinyl-CoA synthetase beta subunit, aconitate hydratase and bifunctional aconitate hydratase 2/2-methylisocitrate dehydratase that are associated to the TCA cycle. Other induced proteins were involved in glycolysis such as the enolase (91% identity with enolase from *Ralstonia solanacearum*) and pyruvate kinase (76% identity with pyruvate kinase from *Cupriavidus taiwanensis* LMG 19424. The ATP synthase beta subunit was also induced in cells grown on *p*-cymene showing 87% identity with the protein from *C*. *taiwanensis* LMG 19424. The induction of the proteins from the energy metabolism suggests that cells require a high energy production during growth on *p*-cymene. Four proteins involved in protein and RNA synthesis were also induced by *p*-cymene. The proteins 72 and 101 were identified as the 30S ribosomal protein S1 (BxeA0982) and the alpha subunit of the DNA-directed RNA polymerase (BxeA0340). *p*-Cymene-induced proteins 39 (BxeA0299) and 86 (BxeA0311) were identified as elongation factors. Proteins involved in aromatic metabolism were also induced by *p*-cymene. BphA2 (BxeC1196) protein is the beta subunit of the biphenyl dioxygenase BphA involved in the initial oxidation of biphenyl and PCBs. 4-diphosphocytidyl-2C-methyl-D-erythritol kinase, an enzyme of the deoxyxylulose phosphate pathway of terpenoid biosynthesis is also induced, showing high identity (57%) with the protein from *R*. *solanacerum* CFBP2957. Other induced proteins are listed in Table 2. The highly induced protein 90 (11.9 fold) showed high identity (51%) to a protein from *Polaromonas* sp. JS666, but its function is unknown. Fig 6 shows the proteins repressed by *p*-cymene. Table 3 listed the repressed proteins, which were ordered into subgroups according to their cellular function. Eight proteins synthesized only in glucose are related to transport, energy metabolism, stress, protein synthesis and regulation. A number of proteins associated to transport and membranes were repressed. Protein 10 was identified as a Fe^+2^ transporter with high identity (67%) to Fe^2+^ transporter from *Achromobacter xylosoxidans* AXX-A (Table 3). Protein 17 (3.5 fold repressed) is an ABC transporter (BxeA0391) that showed 55% identity with the ABC transporter associated to toluene tolerance from *C*. *metallidurans* CH34. Proteins (≥2 fold repressed) 17, 27, 47, 136 and 140 (BxeA0391, BxeB2189, BxeA0050, BxeA0597, BxeA3451) were identified as ABC transporter proteins. These polypeptides are predicted to be involved in toluene tolerance (17), amino acid transport (27), sugar transport (140). Polypeptides 17, 47 and 136 are periplasmic ligand binding proteins. The 136 (BxeA0597) and 140 (BxeA3451) proteins were only observed in glucose-grown cells. Proteins 12 (BxeA1544) and 28 (BxeA0762) involved in PHA synthesis and *β*-oxidation were repressed by *p*-cymene. Protein 124 (BxeA3816) that was only observed in glucose showed 91% identity with co-chaperonine GroES from *R*. *pickettii* 12D. Some proteins from the energy metabolism were repressed in *p*-cymene such as glyceraldehyde-3-phosphate dehydrogenase (BxeA0566), inorganic pyrophosphodiesterase (BxeA3400) and bifunctional 4-hydroxy-2-oxoglutarate aldolase/ 2-dehydro-3-deoxyphosphogluconate aldolase (BxeA0590). The 4-hydroxy-2-oxoglutarate aldolase (BxeA0590) that was only induced in glucose-grown cells is involved in pentose phosphate pathway. Two proteins that were repressed by *p*-cymene are involved in the metabolism of aromatic compounds such as anthranilate isomerase acting on peripheral anthranilate catabolic pathway and BphB protein involved on the degradation of biphenyl and PCBs. Additional proteins repressed by *p*-cymene were grouped within diverse cellular functions. Interestingly, a diguanylate cyclase/phosphodiesterase that possesses high identity (37%) with diguanylate cyclase/phosphodiesterase from *Allochoromatium vinosum* DSM180 was repressed by *p*-cymene (1.7 fold). This protein is involved in the biosynthesis of the second messenger di-cGMP, which is involved in biofilm synthesis. Therefore, the effect of *p*-cymene on biofilm formation by strain LB400 was studied (S1 Fig, Fig 7). Increased biofilm formation by glucose-grown cells than by *p*-cymene-grown cells was observed using crystal violet staining in microplates (S1 Fig). The Fig 7 shows the confocal micrographs of LB400 cells grown on *p*-cymene (D, E and F) and on glucose (A, B and C). The dead cells were stained with IP (B and E) and total cells were stained with SYTO9 (A and D). C and F micrographs show the live and dead cells composition of the biofilm. The biofilm is synthesized with glucose-grown cells showed a ten times higher density than the biofilm of *p*-cymene-grown cells. In addition, viable cells (CFU/mL) in the biofilm free of planktonic cells were determined. The glucose biofilm is formed by 2.7 ×10^9^ CFU/mL while the *p*-cymene biofilm is formed by 5.1 × 10^7^ CFU/mL. These results indicate a decrease of almost two orders of magnitude in biofilm formation in strain LB400 by *p*-cymene-grown cells compared to glucose-grown cells.

## Discussion

### Functionality of *p*-cymene and *p*-cumate catabolic pathways in *B*. *xenovorans* LB400

The *cym*/*cmt* gene cluster that encodes the proteins of the *p*-cymene and 2,3-dihydroxy-*p*-cumate catabolic pathways is located on the LB400 major chromosome, which encodes main central functions and is under stronger selection than the other two replicons [14]. The organization of the *cym*/*cmt* gene cluster from strain LB400 is similar to the *cym*/*cmt* gene clusters from *p*-cymene/*p*-cumate-degraders *P*. *putida* strains F1 and KL47 and *Rhodopseudomonas palustris* [5,6,8]. In contrast to *P*. *putida* F1, the *cymR* regulator gene from *B*. *xenovorans* LB400 is located between *cmt* genes and not at the end of the *cym*/*cmt* gene cluster. The *cym* and *cmt* genes of the *p*-cymene-degrader *P*. *putida* KL47 are constitutively expressed due to the absence of a functional regulator gene [7]. *P*. *putida* F1 *cymR* gene mutant showed also a constitutive expression of the *cym/cmt* genes [11]. The *cymR* gene product from strain LB400 is probably the transcriptional repressor of *p*-cymene and *p*-cumate catabolic pathways. The expression of the *cymAb* and *cmtAb* genes in LB400 cells grown in *p*-cymene, but not in cells grown in glucose or succinate, and the additional expression of the *cmtAb* gene in *p*-cumate-grown cells suggest that both *p*-cymene and *p*-cumate may induce the expression of these genes and may be potential effectors for the transcriptional regulator CymR. Interestingly, the *cymAb* gene expression is only induced by *p*-cymene. In strain *P*. *putida* F1, *p*-cumate is the sole effector for the transcriptional repressor CymR from the *p*-cymene and *p*-cumate pathways [6,26]. Recombinant *E*. *coli* strains carrying promotor sequences and *cmtAaAbAccmtC*, *cmtC* or *cymB* genes from *P*. *putida* F1 in absence or presence of a plasmid encoding the *cymR* gene, indicated that CymB and CmtC enzymatic activities are present in the absence of the CymR regulator, and that the transcriptional repressor CymR only blocked the enzymes CymB and CmtC in presence of *p*-cumate but not in presence of *p*-cymene [6]. LB400 growth in *p*-cymene indicates that the absence of the *cymE* gene in its genome is not critical for *p*-cymene metabolism. CymE protein has been described as an acetyl-CoA synthetase whose role in *p*-cymene metabolism remains unknown [6]. The growth on *p*-cymene and on *p*-cumate, suggests that strain LB400 is degrading *p*-cymene through the *p*-cumate route and the 2,3-dihydroxy-*p*-cumate central pathway into TCA cycle precursors, providing energy power to the metabolic processes. LB400 growth on diverse aromatic compounds including 3- and 4-hydroxybenzoate, 3- and 4-hydroxyphenylacetate, benzoate, biphenyl, phenylacetate and 2-aminophenol has been reported [14–18,27]. The *p*-cymene peripheral pathway starts with the monooxygenation of *p*-cymene by CymA in presence of oxygen and NADH. CymB and CymC use as substrate NAD^+^ generated at the first reaction for the two next steps (Fig 2). LB400 growth on *p*-cumate indicates that *p*-cumate is degraded into TCA cycle precursors (Fig 2). LB400 growth on *p*-cymene and *p*-cumate, the *cymAb* and *cmtAb* genes transcriptional in *p*-cymene-grown cells, and *p*-cumate dioxygenase CmtA enzymatic activity strongly suggest that *p*-cymene is degraded into 2,3-dihydroxy-*p*-cumate via the aerobic classic pathway encoded by the *cymAaAbBC* gene cluster and *cmtAB* genes from strain LB400. LB400 *cmtAb* gene expression is induced by *p*-cumate, which has been described as the only effector of the *p*-cumate pathway in *P*. *putida* F1 [28]. The results of our study suggest that the transcriptional regulator CymR regulates the *p*-cymene catabolic pathway from *B*. *xenovorans* LB400 using as inducer *p*-cymene. In addition, the transcriptional regulator CymR regulates the LB400 *p*-cumate catabolic pathway using as inducer *p*-cumate and probably also *p*-cymene. The proteomic analysis of LB400 *p*-cymene-grown cells compared to glucose-grown cells showed an increase of CymD protein level, which also suggests the regulation of the *cym* genes by *p*-cymene. The results of this study demonstrate the functionality of the classic aerobic *p*-cymene and *p*-cumate pathways from *B*. *xenovorans* LB400.

### LB400 global response to *p*-cymene

**Fig 8:**
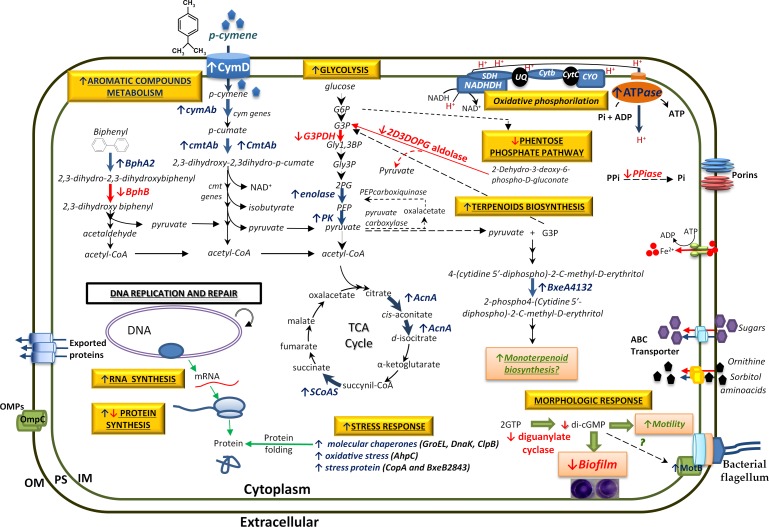
Schematic metabolic network activated by *p*-cymene in *B*. *xenovorans* LB400. Capital and small letters indicate protein and genes, respectively. Red arrow indicates reduced protein synthesis or gene expression. Blue arrow indicates an increase in enzymatic activity, protein level or gene expression. Main *p*-cymene induced metabolic and physiological changes are highlighted by yellow boxes. OM, outer membrane; PS, periplasmic space; IM, inner membrane. BphA2, biphenyl 2,3-dioxygenase beta subunit; BphB, biphenyl dihydrodiol dehydrogenase; CmtAb, *p*-cumate dioxygenase large subunit; G3PDH, glyceraldehyde 3-phosphate dehydrogenase; PK, pyruvate kinase; AcnA, aconitate hydratase; SCoAS, Succinyl-CoA synthetase; NADHDH, NADH dehydrogenase; SDH, Succinate dehydrogenase (Complex II); UQ, ubiquinone; Cytb, ubiquinol-cytochrome C (Complex III); CytC, cytochrome C; Cyo, cytochrome C oxidase (Complex IV); ATPase, ATP synthase; PPiase, inorganic pyrophosphatase; BxeA4132, 4-diphosphocytidyl-2-methyl-D-erythritol kinase.

Fig 8 illustrates the metabolic network activated by *p*-cymene in *B*. *xenovorans* LB400. An aromatic compound is for bacteria not only a carbon and energy source, but may be also a major stress factor. Aromatic compounds may cause damage at the cell membranes, nucleic acids and specific proteins, and may cause cell death [15,20,21,29–31]. The antagonistic effects of an aromatic compound that is a nutrient and also a toxic molecule determines that aromatic-degrading bacteria respond to the presence of an aromatic substrate adjusting their basic vital programs such as the metabolic, the stress and the morphological programs [30]. The proteomic analysis showed diverse *p*-cymene-induced proteins that are part of these three programs.

#### Morphological and stress response to *p*-cymene

LB400 cells incubated in presence of *p*-cymene and *p*-cumate showed fuzzy outer and inner membranes. Due to the hydrophobic character of *p*-cymene and *p*-cumate, an accumulation in membranes is expected. Changes in membrane structure by aromatic compounds have been described in diverse microorganisms including *B*. *xenovorans* LB400 [15,20,21,29,32]. Previously, it was reported that LB400 cells exposed to biphenyl and 4-chlorobiphenyl showed fuzzy membranes [15]. In *P*. *putida* DOT-T1 the presence of toluene damaged inner and outer membranes [33]. Proteomic analyses indicate that LB400 *p*-cymene-grown cells showed altered expression levels of a number of membrane proteins involved in the cell envelope and transport, which correlate with morphologic changes at the membranes. In addition, *p*-cumate caused significant bacterial cell lysis, suggesting that the damage occurred at the cytoplasmic membrane. Interestingly, *p*-cymene cause minor cell lysis than *p*-cumate. The differential effects of *p*-cymene and *p*-cumate could be due to their different hydrophobicity. Hydrophobicity of an organic compound is expressed in terms of octanol-water partition coefficient (P_OW_). Log P_OW_ is inversely correlated with the toxicity of (chloro)biphenyls [21]. Log P_OW_ is higher for *p*-cymene (Log P_OW_ 4.10) than for *p*-cumate (Log P_OW_ 2.99) [34,35]. Therefore, *p*-cumate is more hydrophilic and, thus, a higher concentration in the aqueous phase is expected, affecting the inner and outer membranes and producing higher cell lysis that *p*-cymene. LB400 cells grown in succinate in presence of *p*-cymene showed membranes damage and the presence in the cytoplasm of dense granules that probably are polyphosphates (polyP) [15,20,36,37]. PolyP are accumulated in bacteria during stressful conditions, and contribute to the resistance to stress generated by heat shock, oxidants, osmotic challenge, antibiotics and UV radiation. In *P*. *aeruginosa* PAO1 impaired polyP synthesis correlates with reduced motility, reduced biofilm development, aberrant quorum sensing and reduced virulence [36,38–41]. LB400 growth on succinate in presence of *p*-cumate probably constitutes a stressful condition. Stress response of *B*. *xenovorans* LB400 to PCBs and their derivatives have also been reported [15,20]. In addition, LB400 expression analyses strongly suggest that succinate repressed the expression of the *cmtAb* and *cymAb* genes, indicating that the carbon source regulation is present in LB400 *p*-cymene catabolic pathway. Succinate exerts a strong effect on *p*-cymene and *p*-cumate catabolic pathways from strain LB400, a phenomenon known as catabolic repression by carbon source. The metabolism of *p*-cymene induce in strain LB400 a cellular stress program, which includes proteins and genes that allow adaptation to sub-optimal growth conditions, defense mechanisms to counteract reactive oxygen species (ROS) and molecular chaperones that are induced by various stress factors such as the heat shock and pH changes. The induction by *p*-cymene of the chaperons GroEL, DnaK and ClpB and the copper oxidase CopA indicates that exposure to *p*-cymene constitutes a stressful condition. CopA is a multicopper oxidase, which oxidize its substrate by accepting electrons at a mononuclear copper center and transferring them to a trinuclear copper center that binds dioxygen following the transfer of four electrons to reduce two water molecules. Phylogenetic analysis showed that LB400 CopA protein is a multi-copper oxidase that is active in copper resistance but not in homeostasis. The organic hydroperoxide reductase Ohr and the alkyl hydroperoxide reductase AhpC were induced in presence of *p*-cymene, which suggest that LB400 cells exposed to *p*-cymene are subjected to oxidative stress. The induction of aconitate hydratase AcnA, which catalyze the reversible isomerization of citrate and isocitrate via *cis*-aconitate, may be associated with the main role of AcnA in a metabolic by-pass for cell maintenance during oxidative stress [42]. In *E*. *coli* it has been shown that AcnA replaces the function of AcnB under oxidative stress [43]. These results indicate that the presence of *p*-cymene constitutes an oxidative stress condition for strain LB400. A high number of outer membrane proteins and transporters were induced during growth on *p*-cymene. Some of these proteins may be associated to detoxification of metabolites from the *p*-cymene degradation. In diverse stress conditions caused by aromatic compounds, outer membrane proteins and transporters are active [15,20]. The induction of these polypeptides could be related to an increase of unfolded or precursor proteins, due to the presence of *p*-cymene. Aromatic compounds may perturb the membranes, generate unfolded envelope proteins and activate the envelope stress response [44,45]. *p*-Cymene also induced elongation factors Tu and G in *B*. *xenovorans* strain LB400. EF-G, which is induced under different stressful conditions including the presence of 4-chlorobiphenyl [15,46–48], is capable to interact with unfolded proteins as a classic molecular chaperone [48]. In addition, bioinformatic analysis indicates that BxeB2035 gene encodes a diguanylate cyclase/phosphodiesterase protein, which is repressed in *p*-cymene-grown cells. Diguanylate cyclase (DGC) synthetizes cyclic diguanosine monophosphate (c-di-GMP) from GTP, whereas phosphodiesterase (PDE) degrades c-di-GMP into pGpG. c-di-GMP is a ubiquitous second messenger that is used by most bacteria to drive the switch between a free-living (planktonic) and a sedentary (biofilm) lifestyle [49,50]. LB400 diguanylate cyclase/phosphodiesterase has the catalytically active GGDEF domain of DGC, and the EAL domain that is critical for the PDE activity [49–52]. In bacteria, in general the diguanylate cyclase/phosphodiesterase has one function either DGC or PDE. However, in *Mycobacterium smegmatis* a bifunctional DGC/PDE enzyme has been reported [50,53]. In this study, LB400 *p*-cymene-grown cells showed a decrease in biofilm formation compared to glucose-grown cells. LB400 proteomic analysis showed a down-regulated BxeB2035 protein, which suggests a decreased diguanylate cyclase activity. The decrease of the DGC protein level in LB400 cells grown on *p*-cymene leads to lower c-di-GMP levels and may increase bacterial motility, which correlates with an increase of motility protein MotB associated to flagellar motor (Fig 8). The role of c-di-GMP in biofilm formation in *Burkholderia* genus was first reported in *B*. *cenocepacia* H111 [54]. In conclusion, LB400 *p*-cymene-grown cells showed reduced biofilm formation and probably possess a higher motility than glucose-grown cells.

#### Metabolic response to *p*-cymene

Several LB400 proteins of the metabolic program were induced by *p*-cymene. The outer membrane protein CymD protein (BxeA3558) associated to *p*-cymene import was induced. *p*-Cymene is internalized into the cytoplasm by CymD in *P*. *putida* F1 [6]. In addition to the increased expression of the catabolic *cymAb* and *cmtAB* genes, the catabolic biphenyl dioxygenase BphA2 protein was also induced by *p*-cymene (Fig 8). On the other side, the induction by *p*-cymene of 4-diphosphocytidyl-2C-methyl-D-erythritol kinase, which is a member of the GHMP kinase family, was observed. 4-diphosphocytidyl-2C-methyl-D-erythritol kinase is an enzyme of the deoxyxylulose phosphate pathway of terpenoid biosynthesis that has been identified in *E*. *coli* and the plant *Solanum lycopersicum*. 4-Diphosphocytidyl-2C-methyl-D-erythritol kinase is an essential enzyme in the non-mevalonate pathway (HMG-CoA reductase pathway) of isopentenyl diphosphate and dimethylallyl diphosphate biosynthesis, catalyzing the single ATP-dependent phosphorylation step. The induction of this protein could be associated with the terpenoid nature of *p*-cymene and its metabolism. We propose that pyruvate, which is the final product of *p*-cymene metabolism, and 3-phosphoglycerate are used by strain LB400 for its terpenoid biosynthesis. An increase of proteins involved in RNA and protein synthesis (DNA-directed RNA polymerase subunit alpha and 30S ribosomal protein S1) by *p*-cymene was also observed, which suggest an increased protein synthesis in cells growing on *p*-cymene. The amino acid metabolism is also affected by *p*-cymene, which suggests the synthesis of diverse accessory reactions required for the degradation pathways and global metabolism such as co-factors and essential metabolites synthesis and reducing power generation/recycling. Diverse proteins of glycolysis, TCA cycle and oxidative phosphorylation were increased by *p*-cymene. Pyruvate kinase, enolase, aconitase AcnA, succinyl-CoA synthetase subunit beta and ATP synthase beta subunit are involved in glycolysis, TCA cycle and ATP production by oxidative phosphorylation (Fig 8). The induction of these proteins involved in the cellular energy production, is probably required for the high-energy demand for the adaptation of strain LB400 to *p*-cymene. The degradation of *p*-cymene and *p*-cumate by strain LB400 activates the basic cellular programs that allow the proper function of cellular metabolism in the presence of aromatic compounds that may cause damage at membranes and oxidative stress as shown in Fig 8. This study suggests that the regulation of protein and RNA synthesis is of crucial importance for the adequate expression of *p*-cymene and *p*-cumate pathways. In addition, this report indicates that to optimize the *p*-cymene/*p*-cumate degradation processes in strain LB400, the enzymatic activities and proteins involved in processes such as stress response, morphological response and metabolic response need to be balanced in order to avoid accumulation of toxic metabolites or by-products such as ROS. Bioinformatic analyses, functional assays, proteomic and transcriptomic studies strongly suggest that the *cym/cmt* genes cluster present in the genome of strain LB400 encode the proteins involved in the catabolism of *p*-cymene and *p*-cumate. The versatile metabolism for aromatic compounds of the model bacterium *B*. *xenovorans* LB400 may be useful to study systems biology and for biotechnological applications such as the bioremediation of aromatic compounds including *p*-cymene and *p*-cumate in polluted environments. In conclusion, this study describes active *p*-cymene and *p*-cumate catabolic pathways in *B*. *xenovorans* LB400, and shows that *p*-cymene activated a stress response and reduced biofilm formation in LB400 cells.

## Supporting Information

S1 Fig Effect of *p*-cymene on *B*. *xenovorans* LB400 biofilm formation in microplates. Cells grown in M9 medium using glucose or *p*-cymene as sole carbon source until exponential phase (Turbidity_600nm_ = 0.6) and washed were resuspended in M9 medium supplemented with glucose (5 mM) or p-cymene (vapor phase). Biofilm qualitative determination was performed after 48 hours of incubation in glucose (A) or p-cymene (B) and staining with 0.1% crystal violet in microplates.
(DOCX)
