# Supplementary material for: p-Cymene Promotes Its Catabolism through the p-Cymene and the p-Cumate Pathways, Activates a Stress Response and Reduces the Biofilm Formation in Burkholderia xenovorans LB400
Source: PLoS One. 2017 Jan 10;12(1):e0169544. doi: 10.1371/journal.pone.0169544 (PMC5224996; doi:10.1371/journal.pone.0169544)
Supplement: S1 Fig — Cells grown in M9 medium using glucose or p-cymene as sole carbon source until exponential phase (Turbidity600nm = 0.6) and washed were resuspended in M9 medium supplemented with glucose (5 mM) or p-cymene (vapor phase). Biofilm qualitative determination was performed after 48 hours of incubation in glucose (A) or p-cymene (B) and staining with 0.1% crystal violet in microplates. (DOCX) [file pone.0169544.s001.docx]

**
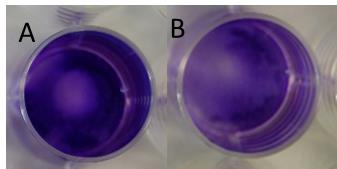
**

**S1 Fig**. **Effect of *p*-cymene on *B. xenovorans* LB400 biofilm formation in microplates.** Cells grown in M9 medium using glucose or *p*-cymene as sole carbon source until exponential phase (Turbidity_600nm_=0.6) and washed were resuspended in M9 medium supplemented with glucose (5 mM) or p-cymene (vapor phase). Biofilm qualitative determination was performed after 48 hours of incubation in glucose (A) or p-cymene (B) and staining with 0.1% crystal violet in microplates.
